# Case Report: A heterozygous loss-of-function variant of the *ERG* gene in a family with vascular pathologies

**DOI:** 10.3389/fcvm.2025.1550523

**Published:** 2025-06-24

**Authors:** Philipp Erhart, Nicola Dikow, Eva M. C. Schwaibold, Susanne Dihlmann, Caspar Grond-Ginsbach, Daniel Körfer, Christian P. Schaaf, Sabrina Oeser, Katrin Hinderhofer, Dittmar Böckler, Jiarna R. Zerella, Hamish S. Scott, Christopher N. Hahn, Felix Marbach

**Affiliations:** ^1^ Department of Vascular and Endovascular Surgery, University Hospital Heidelberg, Heidelberg, Germany; ^2^ Institute of Human Genetics, Heidelberg University, Heidelberg, Germany; ^3^ Molecular Pathology Research Laboratory, Centre for Cancer Biology, SA Pathology and University of South Australia, Adelaide, SA, Australia; ^4^ Adelaide Medical School, University of Adelaide, Adelaide, SA, Australia; ^5^ Institute of Human Genetics, Medical Faculty, University of Bonn, Bonn, Germany

**Keywords:** multiple arterial aneurysms, *ERG*, abdominal aortic aneurysm, loss-of-function variant, haploinsufficiency

## Abstract

**Background:**

The transcription factor *ERG* (erythroblast transformation-specific-related gene) has been identified as a key regulator of vascular function by suppressing inflammation in endothelial cells (ECs). Dysregulation of *ERG* due to genetic risk variants is linked to chronic inflammation in conditions such as atherosclerosis and aortic aneurysms.

**Case presentation:**

This research work investigates the role of the *ERG* gene in the development of a systemic arterial aneurysm manifestation. Given the previous implication of *ERG* in vascular development, we now report a loss-of-function variant (Leu212*) in the *ERG* gene, segregating in a family with vascular pathologies. Multiple arterial aneurysms were observed in one family member, and early onset of vascular-associated stroke in another individual carrying the familial *ERG* variant. Histological analysis of arterial aneurysm specimen showed comparable expression of ERG in endothelial cells of the vasa vasorum in samples from the patient and controls.

**Conclusion:**

Our report discusses the possibility that loss-of-function variants in *ERG* may act as a risk factor for arterial disease.

## 1 Introduction

The etiology of arterial aneurysms is mostly multifactorial, and both genetic and environmental factors contribute to their development. Previous research to identify genetic risk loci for aneurysm development through genome-wide association studies identified several loci, including the SNP NM_182918.4: c.19-2286G>T (rs2836411) in the *ERG* gene (1). *ERG* (erythroblast transformation-specific-related gene) encodes a transcriptional regulator protein, which influences endothelial homeostasis, angiogenesis, cell proliferation, inflammation, and apoptosis. ChIP-seq analysis in human umbilical vein endothelial cells demonstrated that the ERG protein binds lineage-specific endothelial super enhancers; promoting endothelial homeostasis, and enrichment of SNPs within these super-enhancers was suspected to contribute to cardiovascular disease and aortic aneurysm formation (2). Investigation of the gene regulatory function demonstrated that the rs2836411 SNP affected *ERG* expression in vascular endothelial cells and enhancer activity contributing to abdominal aortic aneurysm formation (3). In a single-cell transcriptome analysis of ascending thoracic aortic aneurysms, expression of *ERG* was found to be decreased in five cell clusters, when compared to healthy controls, suggesting a role of ERG in the maintenance of the aortic wall (4). Population genetics and animal models support a vascular disease-association of *ERG* in humans: In the gnomAD v4.1.0 population database, containing 730,947 exomes and 76,215 genomes the loss-of-function observed/expected upper bound fraction (LOEUF) score of *ERG* is 0.234 with 5 observed LoF variants vs. 44.8 expected variants, suggesting evolutionary selection against *ERG* haploinsufficiency. In mouse models, biallelic knockout of the murine homolog *Erg* resulted in embryonic death due to “occlusion and narrowing of pulmonary venules, pancytopenia, and variable pulmonary capillary hemorrhage” (5). Endothelial-specific knockout of *Erg* led to increased embryonic lethality with disorganized blood vessels in knockout embryos (6), indicating a pivotal role of the gene during embryonic development in mice. *ERG* is specifically expressed in endothelial cells promoting anti-inflammatory effects via repression of inflammatory genes such as interleukin-8 (7). Endothelial barrier function is also mediated by claudin 5 (*CLDN5*) a downstream target of ERG. *ERG* knockdown was reported to result in an increase of endothelial gap formation and permeability (8). We now report a family with a LoF variant in ERG with multiple aneurysms and early-onset vascular-associated stroke. The 52-year-old male index patient of this case report presented with an asymptomatic abdominal aortic aneurysm which was diagnosed by a routine examination. Further investigation revealed multiple arterial aneurysms. Physical examination of the patient was not suggestive of a known connective tissue disorder (Beighton Score 0, Ghent Score 0).

## 2 Case presentation

**Figure 1:**
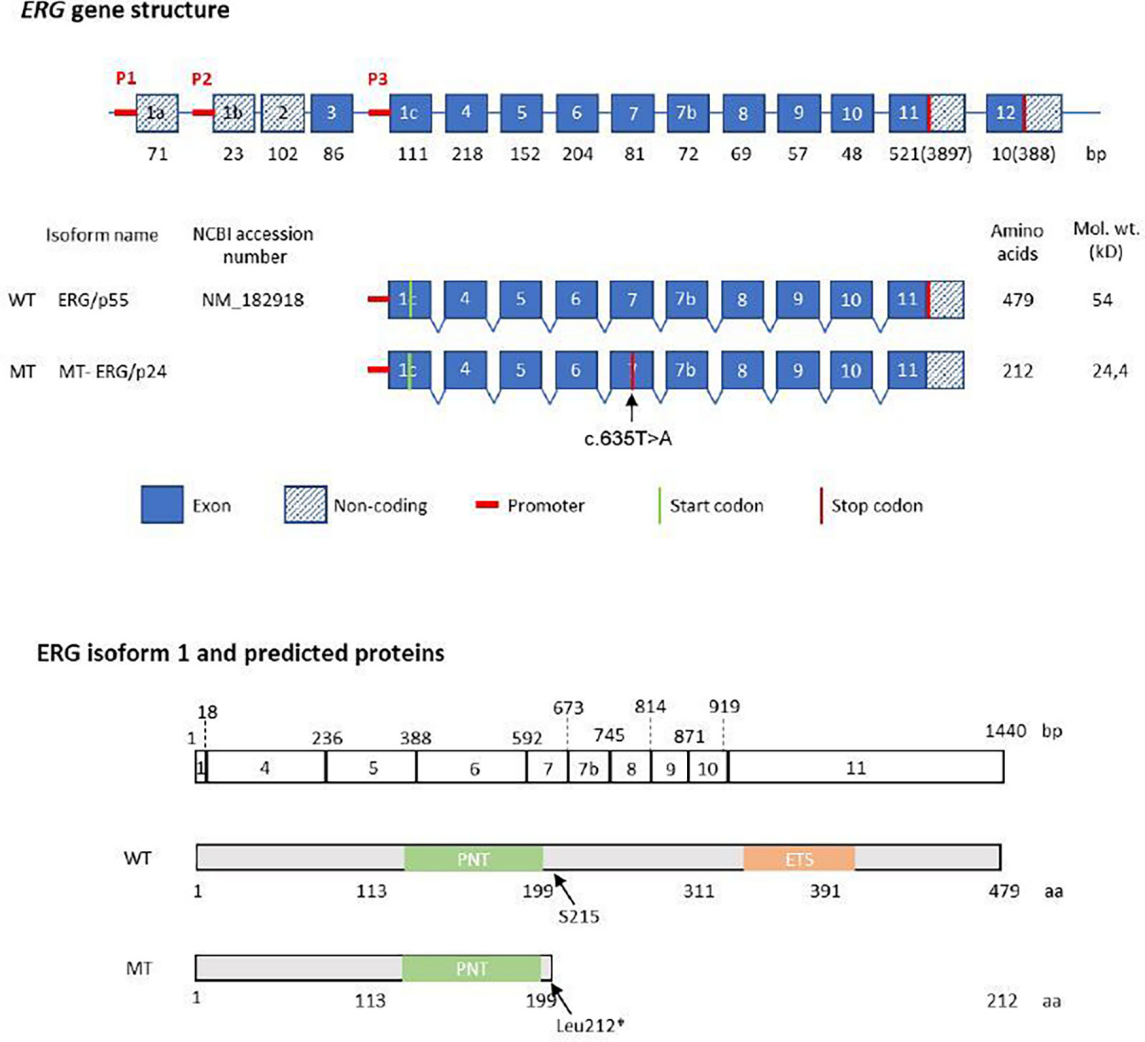
Structure of the human *ERG* gene and protein. Structure of the human *ERG* gene and protein p55, according to the nomenclature suggested by Zammarchi (9). Top: schematic representation of *ERG* coding exons (encoding eight isoforms), shown with their size in base pairs (bp) below each exon. Middle: genomic region of *ERG* isoform p55*,* showing position of the affected exon and the STOP codon, predicted size of the WT and mutant allele. Below: the ERG/p55 exon structure and nucleotide length (in base pairs) is aligned with the predicted protein sequence showing the amino acid position of the main protein domains. PNT (pointed domain), ETS (ETS DNA-binding domain). The phosphorylated serine residue at position 215 is indicated by an arrow.

**Figure 2:**
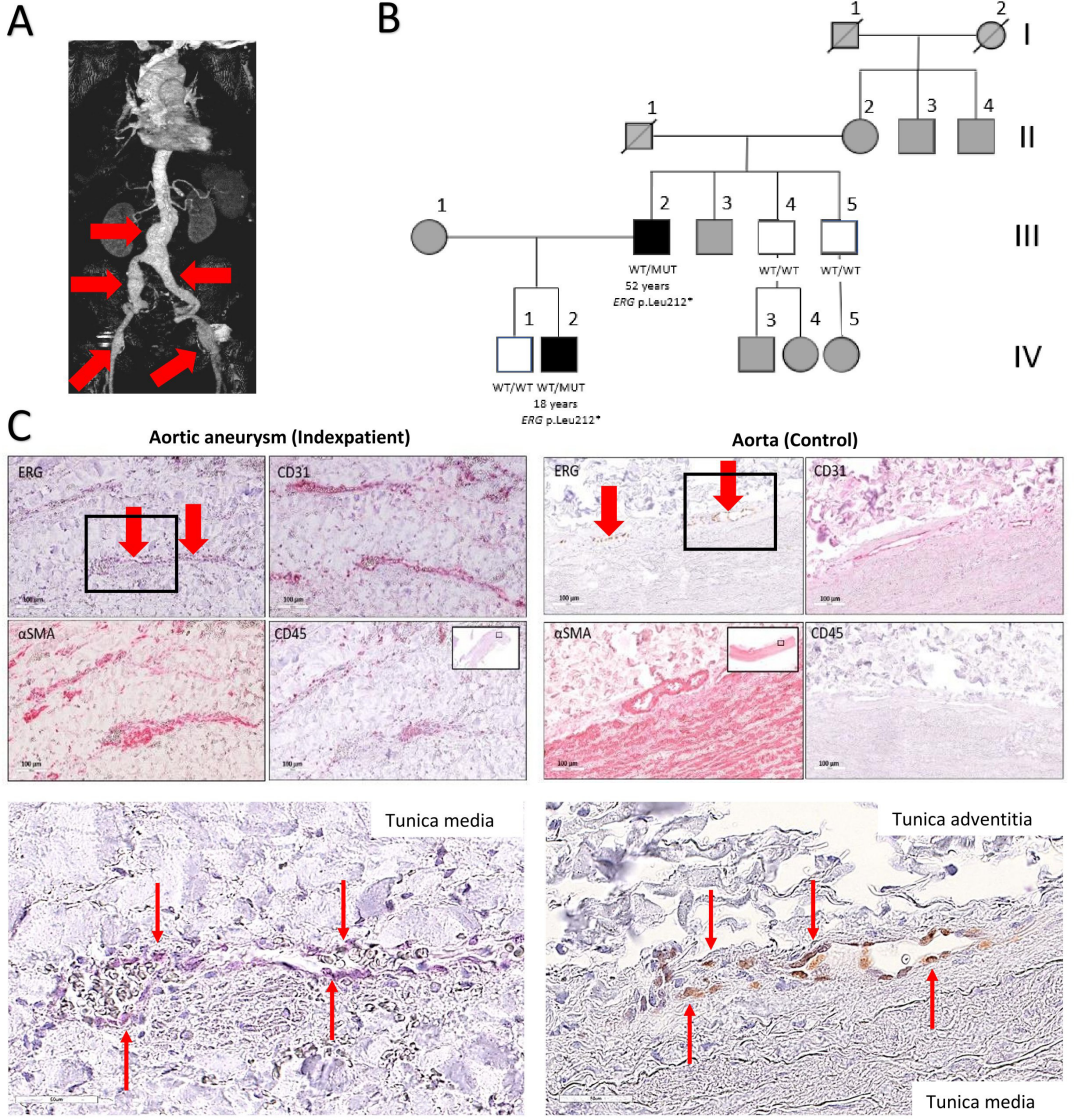
Multiple aneurysms in an individual with *ERG* haploinsufficiency. **(A)** 3-dimensional reconstruction of the computed tomography angiography of the index patient revealed multiple aneurysm formation. Red arrows indicate aneurysmatic vascular segments. **(B)** Family pedigree including the index patient with multiple arterial aneurysms (age 52 years) and an affected son (18 years) with cervical artery dissection and the same *ERG* gene variant. Other family members were not available for Sanger sequencing analysis. WT/WT: wild type homozygous, WT/MUT: heterozygous variant, white coloration: investigated without phenotype, black coloration: affected individual, grey: clinically not investigated. **(C)** Immunohistochemically and magnification (indicated by black rectangles) stained sections comparing the abdominal aortic aneurysm (AAA) of the index patient and a healthy control aorta. Staining of sequential histological sections with antibodies for ERG, CD31, αSMA and CD45 revealed expression of ERG exclusively in individual endothelial cells (red arrows) of the vasa vasorum in a similar extent and pattern as in healthy aortas. Scale bars: 100 μm (top) and 50 μm (magnification sections). Red arrows indicate ERG-positive cells in the area of the tunica media (index patient) and in the transition zone between the tunica media and tunica adventitia (control sample).

As part of a genetic consultation at our university hospital, a primary whole exome sequencing (WES) instead of a gene panel for vascular diseases was performed due to the multiple vascular manifestations. The results and variants were assessed for rarity and functionality. WES revealed a heterozygous germline variant in *ERG* (NM_182918.4:c.635T>A, p.(Leu212*) in the peripheral blood of the patient. ClinGen (Clinical Genome Resource database) has not yet published curations for *ERG* and the variant is absent from large population databases including the gnomAD (Genome Aggregation Database) v4.1.0 dataset of 807.162 samples. The variant is located in the fifth exon of the 10-exon transcript, and is expected to result in heterozygous loss of function (LoF) due to nonsense-mediated decay of mutant RNA. The variant location within the *ERG* gene and protein structure are illustrated in Figure 1. An additional heterozygous prothrombin variant [G20210A, a polymorphism associated with an increased risk of venous thrombotic events (11)] was found but no other pathogenic variants in genes associated with arterial aneurysms were detected. The patient's arterial aneurysm diameters measured 53 mm of the abdominal aorta (AAA), 50 mm on the right and 52 mm on the left common iliac arteries, 20 mm on the right and 40 mm on the left common femoral arteries, 42 mm on the right and 36 mm on the left popliteal arteries, 6 mm on the anterior cerebral artery and 4 mm on the basilar artery. Open surgical procedures were performed to prevent rupture and embolism in all aneurysms except the right femoral and intracranial arteries which are under radiologic surveillance to detect aneurysm progression. Despite cardiovascular risk factors such as hypertension and hypercholesterinemia, the computed tomography angiography (Figure 2A) revealed only minimal arteriosclerotic vascular wall changes that could have contributed to the formation of aneurysms. Other routine angiological examinations, including carotid duplex sonography and ankle-brachial measurement, were unremarkable and excluded early atherosclerotic vascular manifestations. Other cardiovascular risk factors, such as diabetes or smoking, were not present in the examined family members. The patient's father had multiple coronary bypass interventions. One of the patient's two sons, who carried the familial *ERG* variant, had an ischemic stroke (apoplexy) at the age of 18 years due to an acute cervical artery occlusion. Phenotypical signs of lymphatic malformations were absent in all family members such as chronic swelling of body parts or other nail and skin changes. Further segregation analysis by Sanger sequencing neither detected the variant in the proband's other (healthy) son, nor in his healthy brothers (aged 46 and 51 years) (see pedigree in Figure 2B). The microsatellite analysis revealed no evidence of non-relationship between the examined samples. In order to detect morphological vascular changes, we performed histological and immunohistochemical staining (Figure 2C) in aortic, iliac and femoral aneurysm samples of the patient and control aneurysm samples. Staining of sequential histological sections with antibodies for ERG, CD31, αSMA and CD45 revealed expression of ERG in endothelial cells of the vasa vasorum to a similar extent and pattern as in control samples. Despite single-cell RNA sequencing demonstrating expression of *ERG* mRNA in cellular subsets of smooth muscle cells, endothelial cells, and fibroblasts in human ascending aortic tissue (4), we could only identify sparse ERG positive staining in endothelial cells.

## 3 Methods

### 3.1 Immunohistochemistry

Tissues were formalin fixed and embedded in paraffin according to standard procedures for conventional histology. Briefly, 4 µm sections were deparaffinized, rehydrated and incubated in 100 mM citrate buffer, pH 6.0 for antigen retrieval. Immunohistochemical staining was performed overnight with the following primary antibodies: rabbit anti-human ERG (# 97249, Cell Signaling Technology); mouse anti-human CD31 (# 3528, Cell Signaling Technology), rabbit anti-human alpha smooth muscle actin (αSMA) (#19245, Cell Signaling Technology), rabbit anti-human CD45 (# 13917, Cell Signaling Technology). CD31, αSMA and CD45 are key markers used to identify endothelial cells, smooth muscle cells, and leukocytes respectively, and their expression patterns can provide insight into vascular remodeling and inflammatory processes regulated by the *ERG* gene pathway in vascular biology. After washing, detection was performed by using the DAKO REAL Detection System Alkaline Phosphatase/RED, rabbit/mouse, AP/RED, rabbit/mouse (Agilent, Santa Clara, CA, USA). All sections were counterstained with hematoxylin and covered with Aquatex (aqueous mounting agent for microscopy, Merck, Sigma Aldrich, Germany). Detailed protocols are available on reasonable request.

### 3.2 Exome sequencing

Blood samples were obtained after signed informed consent. Exome sequencing was performed using a SureSelect Human All Exon V7-Kit (Agilent, 48.2 Mb design size) for enrichment and a HiSeq 4000/NovaSeq System (Illumina, San Diego, California). Reads were aligned to the UCSC human reference assembly (GRCh38) with BWA. Single nucleotide variants (SNV), small insertions and deletions were detected using GATK. Variants were analyzed in the in-house exome variant analysis database (VarViewS). Variants with a minor allele frequency (MAF) in gnomAD <1% and our in-house database, respectively, that yielded at least one (likely) pathogenic score using different in-silico prediction tools, were assessed. Additionally, the databases ClinVar and HGMD® Professional were reviewed for assessment. Sanger sequencing was performed to confirm the *ERG* variant in the index patient and to exclude the variant in the relatives mentioned below.

### 3.3 Relative mRNA expression of *ERG* and endothelial *ERG* target genes

**Figure 3:**
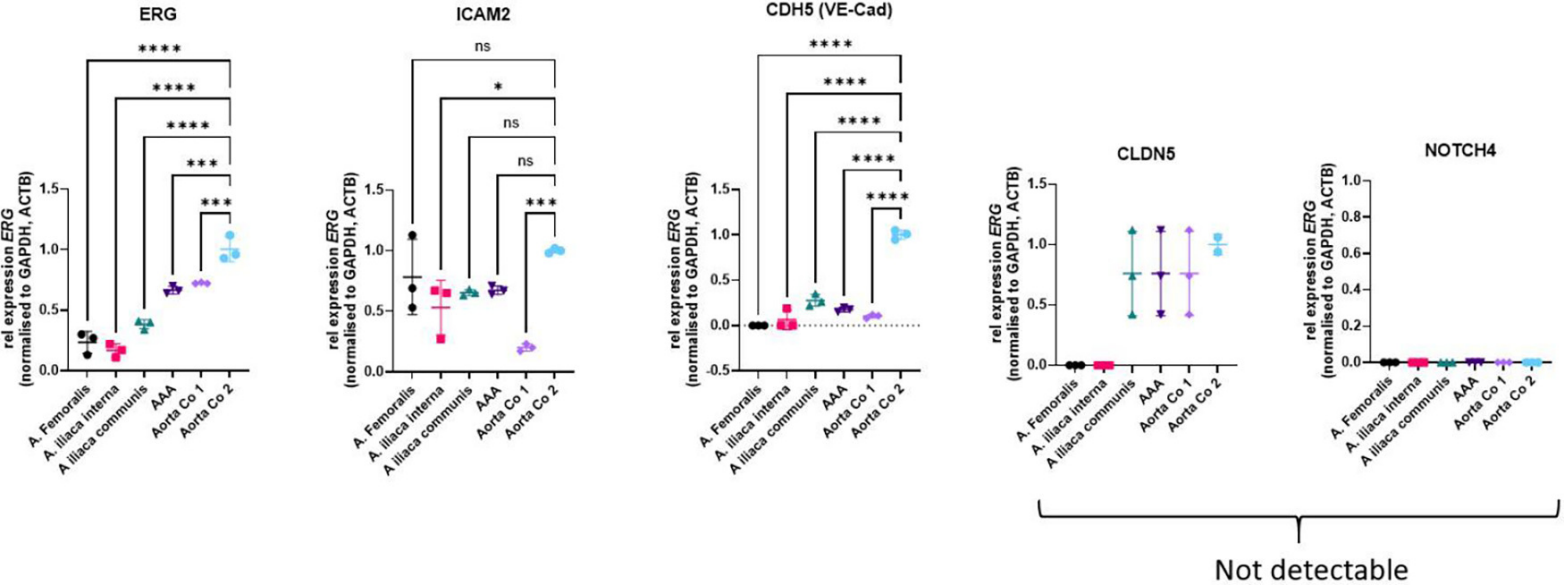
Relative mRNA expression of *ERG* and endothelial *ERG* target genes in aneurysmatic and control aortic tissues. Data are shown as the mean with SD of three technical replicates. Statistical analysis was performed by ordinary one-way ANOVA with Dunnett's multiple comparison test. *: *P* < 0.05; ***: *P* < 0.001; ****: *P* < 0.0001. ACTB, actin beta; Aorta Co1 and Aorta Co2, control aortic specimen 1 and 2; ICAM2, intercellular adhesion molecule 2; CDH5, cadherin-5; CLDN5, claudin-5; GADPH, glyceraldehyde-3-phosphate dehydrogenase; ns, not significant; NOTCH4, neurogenic locus notch homolog 4; VE-Cad, vascular endothelial (VE)-cadherin.

Four fresh frozen biopsies from the individual III/2 were ground with pestle and mortar in liquid nitrogen for RNA extraction using the RNeasy Fibrous Tissue Mini Kit (Qiagen, Hilden Germany) according to the manufacturer's instructions. These biopsies were obtained from surgical aneurysm procedures including the femoral-, internal iliac-, common iliac arteries and the abdominal aorta. Two control samples of human non-diseased aortic tissue (Aorta Co 1 and Co2) were obtained from organ donors stored in the vascular biobank Heidelberg (VBBH) and used for comparison. Real-time RT-PCR was performed by using primers specific for the *ERG* and four of its genetic targets, *ICAM2, CDH5, CLDN5* and *NOTCH4* (Figure 3). Samples were loaded onto 96-well PCR plates and analysed in a StepOnePlus real time PCR System. Quantitative analysis of gene expression was performed relative to expression of GAPDH and ACTB mRNA in corresponding samples by using the 2^−DDCt^ method.

## 4 Discussion and literature review

The *ERG* gene encodes a transcription factor that play a critical role in the endothelial cell function, vascular integrity, and angiogenesis. Mutations or dysregulation of this gene can disrupt downstream pathways, such as the regulation of VEGF (vascular endothelial growth factor) signalling impairing endothelial proliferation, migration and survival (12). Another major downstream pathway regulated by *ERG* is TGF-β (transforming growth factor-beta) signalling controlling endothelial-to-mesenchymal transition leading to fibrosis or atherosclerosis (13). Additionally, inflammatory processes are modulated by *ERG*, such as NF-κB activity and expression of adhesion molecules (ICAM-1, VCAM-1) involved in leukocyte infiltration and chronic inflammatory processes (14). Furthermore, vascular relaxation and increased vascular resistance are disrupted by *ERG* dysfunction via the eNOS (endothelial nitric oxide synthase) cascade (10). Endothelial dysfunction may lead to impaired anti-thrombotic protection resulting in thrombosis and coagulopathy (15) represents an independent risk factor for multiple arterial aneurysms and dissection (16). Given the previous implication of *ERG* in vascular development, and endothelial homeostasis, and the rarity of heterozygous LoF germline variants in the general population, a connection between the familial LoF variant in *ERG* and the vascular pathologies observed in the patient and his son appears plausible despite the unexplained phenotypic variability of vascular diseases. To date, two distinct phenotypes have been reported in individuals with *ERG* haploinsufficiency. A rare variant association study of 77,539 individual genomes found different germline frameshift variants of *ERG* in patients with primary lymphedema from four different families (17). Two of the variants found in lymphedema patients were predicted to result in nonsense-mediated decay, while two others located in the last exon potentially resulted in a truncated or elongated protein. Another group functionally characterized 10 heterozygous germline *ERG* variants identified in a cohort of patients with cytopenia and/or hematological malignancies, demonstrating partial or complete LoF in 8 of the reported missense variants. Some patients with lymphatic malformations have been reported to carry abnormally extended *ERG* proteins (8). As a limitation this study did not perform functional analysis. Another *de novo ERG* variant most likely resulting in nonsense-mediated decay (Tyr372*), was reported in a patient with congenital pancytopenia and bone marrow failure (18). Neither lymphedema, nor hematological disorders, were reported in our pedigree, which could be explained by incomplete penetrance of both phenotypes. We now suggest a connection of *ERG* haploinsufficiency to a third phenotype, i.e., arterial pathologies.

In this case report cryo-conserved specimen were used to perform relative mRNA expression of *ERG* and endothelial *ERG* target genes in aortic and control aortic tissues. The significance of the results is limited, as only a few ERG-positive cells were present in the endothelial layer. Additionally, the analysis is confounded by the presence of other vascular wall cells, such as smooth muscle cells, adventitial fibroblasts, and leukocytes. Therefore, these results cannot be used for definitive conclusions, and isolated endothelial cells should be used to perform ERG-specific expression analysis.

The challenges of investigating the functional consequences of haploinsufficiency of a given gene are steep, as the utility of biallelic knockout models is questionable, and tissue-specific expression alone is not indicative of relevant functional defects. Additionally, evolutionary selection against LoF variants, as observed in the case of *ERG*, is not probative for a measurable postnatal phenotype. A possible selection bias may occur entirely prenatally, and the loss of reproductive fitness required to significantly skew population data against a variant type can be much smaller than expected: An estimation based on the ExAC database of 60,706 human exomes, which focused on selective effects of protein truncating variants (PTVs), found a mean fitness loss of only ∼0.5% for all PTVs, although with a broad distribution (19). The authors proposed that even small selective effects of PTVs could have disproportionate impacts after a sufficiently large number of generations.

*ERG* can be considered as a potential candidate gene, i.e., a gene of unknown significance (GUS), in the context of arterial aneurysms. Within the ACMG classification system, any potentially causative variant in a GUS is classified as a variant of unknown significance (VUS) (20). The question then remains whether the reported disease associations of LoF variants in *ERG* amount to true Mendelian disorders, or whether ERG haploinsufficiency may contribute, as a risk factor, to different human phenotypes in conjunction with other (common) variants. The answer to this question will require the compilation and characterization of additional individuals with germline *ERG* variants. The authors actively seek additional cohorts and collaborations to collect further pathogenic *ERG* variants. Researchers are encouraged to contact the corresponding author.

## Data Availability

The data and materials used in this study are available from the corresponding author upon reasonable request.
